# Molecular screening of piroplasms and Anaplasmataceae agents in *Hyalomma dromedarii* ticks from camels over different seasons in Egypt

**DOI:** 10.1007/s10493-024-00957-w

**Published:** 2024-09-25

**Authors:** Bassma S. M. Elsawy, Hoda S. M. Abdel-Ghany, Heba F. Alzan, Sobhy Abdel-Shafy, Yasser E. Shahein

**Affiliations:** 1 Parasitology and Animal Diseases Department, Veterinary Research Institute, National Research Centre, Dokki, 12622 Giza Egypt; 2 Ticks and Tick-Borne Diseases Research Unit, Veterinary Research Institute, National Research Centre, Dokki, 12622 Giza Egypt; 3 Molecular Biology Department, Biotechnology Research Institute, National Research Centre, Giza 12622 Dokki, Egypt

**Keywords:** Anaplasmosis, Camel, *Hyalomma*, PCR, Piroplasmosis, Sequencing, *Wolbachia*

## Abstract

Piroplasmosis, a disease of domestic and wild animals, is caused by tick-borne protozoa of the genera *Babesia* and *Theileria*, while anaplasmosis is caused by tick-borne bacteria of genera *Anaplasma*. *Hyalomma dromedarii* is the most dominant tick species infesting camels in Egypt and act as a vector of piroplasms, *Anaplasma*, *Rickettsia* and *Ehrlichia* spp. The available information concerning the detection of these pathogens in *H. dromedarii* infesting camels is limited. The present study aimed to evaluate the status of these pathogens in *H. dromedarii* ticks over four seasons of a year, in addition to investigate the infections of piroplasms and Anaplasmataceae besides their genetic diversity starting from June 2021 till April 2022. A total of 275 semi-engorged females of *H. dromedarii* were collected from different slaughtered camels, Toukh city slaughterhouse then investigated by Polymerase Chain Reaction (PCR) to detect piroplasms (*Babesia* spp., *Theileria* spp.) and Anaplasmataceae DNA targeting *18 S rRNA* and *16 S rRNA* genes, respectively followed by sequencing and phylogenetic analyses. Overall, piroplasms were detected in 38 ticks (13.8%), *Babesia* spp. was detected in 35 ticks (12.7%), while *Theileria* spp. was detected in one tick (0.4%). Anaplasmataceae was detected in 57 ticks (20.7%). Mixed infections of piroplasms and Anaplasmataceae were detected in 13 ticks (5%). Single infection either with piroplasms or Anaplasmataceae was detected in 25 (9%) and 44 (16%) ticks, respectively. The highest monthly rate of piroplasms was in April (spring) and Anaplasmataceae was in July (summer). Sequence analysis revealed that *Babesia bigemina*,* Wolbachia* spp. and *Anaplasma marginale* are the most dominant species in the examined tick samples. To the best of our knowledge, this study confirms the presence of *B. bigemina*,* Wolbachia* spp. and *A. marginale* in *H. dromedarii* in Egypt by sequencing.

## Introduction

Ticks are obligate blood feeders, hematophagous arthropods that parasitize broadly on several vertebrate hosts, including wild and domestic animals and humans (Kapo et al. 2024; Nasirian 2024). As a significant vector for vector-borne pathogens, ticks come in second place to mosquitoes (Minjauw and McLeod 2003; Chhillar et al. 2014). Pyemia, anemia, toxicosis, and paralysis are among the direct effects of tick infestations on hosts, with a cumulative estimated loss of over $500 million per year worldwide (Minjauw and McLeod 2003; Chhillar et al. 2014). Ticks and tick-borne pathogens (TBPs) are the main obstacles that hinder the productivity of livestock globally, especially in the developing countries. Worldwide losses totaling between $22 and $30 billion are attributed to the management of ticks and TBPs (Minjauw and McLeod 2003; Kumar et al. 2020).

The total number of camels population recorded in Egypt was 119,885 according to the statistics of FAO 2021 (Ashour and Abdel-Rahman 2022). Recently, it was reported that there is an increase in camel importation to Egypt from different African countries (Elsawy et al. 2023). Camels are mostly infested by *Hyalomma* spp which are known as vectors of piroplasms and Anaplasmatacae (Alsarraf et al. 2017; Alanazi et al. 2019, 2020; Getange et al. 2021). The tick-borne piroplasms are hemoprotozoan pathogens, which are caused by parasites belonging to the genera *Babesia* and *Theileria* (Zanet et al. 2014). Anaplasmatacae are gram negative, obligate intracellular bacteria including the genus *Anaplasma* (Dumler et al. 2001). In most infected animal cases with both pathogens, the clinical manifestations appear in the form of anemia, icterus, hemoglobinuria, fever and risk of mortality leading to considerable economic losses (Abdelbaset et al. 2022).

*Hyalomma dromedarii* (*H. dromedarii*) is the most prevalent tick species on camels in Egypt, whether they are farmed or imported. It is a two- host tick and can parasitize various domestic animals (El-Kammah et al. 2001; Mumcuoglu et al. 2022; Elati et al. 2024). Humans are rarely bitten by *H. dromedarii* ticks (Mumcuoglu et al. 2022). *H. dromedarii* can transmit viruses such as the Crimean–Congo hemorrhagic fever virus (Rodriguez et al. 1997), Dera–Ghazi–Khan virus, Dhori virus (Hoogstraal et al. 1981), and Kadam virus (Wood et al. 1982). In addition, *H. dromedarii* is a vector of protozoan pathogens, including *Theileria camelensis* and *T. annulata* (Hoogstraal et al. 1981), and bacterial pathogens such as Q fever, *Coxiella burnetii* (Bazlikova et al. 1984; Abdullah et al. 2018) and spotted fever rickettsia (Lange et al. 1992; Abdel-Shafy et al. 2012).

The molecular approach is considered the most reliable and sensitive tool to detect pathogen DNA not only in the acute phase of infection but also in the persistent stages where parasitemia is undetectable by the microscopic approach (Rosales et al. 2013; Abdel-Shafy et al. 2022). Therefore, this study aimed to examine semi-engorged females of *H*. *dromedarii* ticks collected from camels in Egypt at four seasons of the year to detect the presence of piroplasms and Anaplasmataceae DNA using PCR and confirmed by sequence analyses.

## Materials and methods

### Collection and preparation of tick samples

A total of 1000 semi-engorged *H. dromedarii* females were collected from different apparently healthy naturally infested camels from Toukh city slaughterhouse (30° 21′ 11.6″ N, 31° 11′ 31.5″E), Qalyubia governorate, Egypt. It is a semi-rural area. Its summers are long, hot, humid, arid, and clear while the winters are cool, dry, and mostly clear. The climate along the course of the year, the temperature typically varies from 10 °C to 36 °C and is rarely below 8 °C or above 40 °C (https://weatherspark.com/y/96919/Average-Weather-in-Toukh-Egypt-Year-Round). Tick pool were collected from each camel neck area using tweezers and kept separately in a special glass tube. Samples were collected monthly, starting from June 2021 till April 2022. Ticks were examined under a stereomicroscope (LEICA DM 750, Russia) and identified according to the key of Walker et al. (2014). Ticks were preserved in microtubes containing 70% ethanol then were thoroughly washed with distilled water and then dried using filter paper (Kumsa et al. 2016). Each tick was longitudinally sectioned into two equal halves using a sterile scalpel; one half was stored at − 20 °C as a backup and the other half was placed in a separate small plastic bag. Then these bags were exposed to liquid nitrogen to facilitate their crushing.

### DNA extraction

Genomic DNA was extracted from 275 tick samples individually [representative number of ticks (*n* = 5) from each camel tick pool from a total of ~ 55 camels, 10 camels per month]. Usually in the winter season the tick infestation is very low to rare, so the number of ticks was chosen depending on the minimum number of ticks that were found on a single camel (which was 5 ticks). Four hundred microliter of lysis buffer and 10 µL of proteinase K (QIAGEN/Germany) were added to each sample and incubated overnight at 56 °C. The lysed products were subsequently centrifuged, then, supernatants were subjected to DNA extraction using DNeasy Blood & Tissue Kits (QIAGEN/Germany) following the manufacturer’s instructions.

### Molecular identification of ticks

To confirm the morphological identification of the camel tick *H. dromedarii*, DNA of tick samples was screened by PCR using a primer pair to amplify the *cytochrome c oxidase subunit-1* (*CO1*) (Table 1). PCR conditions were applied according to Abdullah et al. (2016).

### Molecular detection of piroplasms

All tick DNA samples were screened by semi nested PCR (nPCR) using primers of *18 S rRNA* gene flanking the hypervariable (V4) region and the primer sequences are listed in Table 1. The external and internal PCR reaction conditions were carried out according to Liu et al. (2016). Camel blood samples tested positive for piroplasms were used as positive control and were adopted according to Mahdy et al. (2023). Negative control (lack of DNA) was also included in each PCR reaction. Products of the internal PCR reactions were subjected to Red Safe DNA gel staining (JH Science, iNtRON Biotechnology, US) of 1.5% agarose gel (Simply, gene direx, Taiwan), and the length of the amplified products was estimated using 100 bp DNA ladder (Bioran, life science, Germany).

#### Conventional PCR (cPCR) for detection of *Babesia/Theileria* spp.

Representative samples, with a strong band, that tested positive to piroplasms were examined by a specific primer targeting a fragment of *Babesia*/*Theileria* spp. *18 S rRNA* gene for confirmation before sequencing. The amplification conditions were applied according to Pereira et al. 2016 (Table 1). This test is performed to exclude ticks’ samples positive with piroplasms other than *Babesia* and *Theileria*.

#### Molecular detection of *Babesia* spp. and *Theileria* spp.

The samples that were identified positive to piroplasms were tested again using individual specific primers for *Babesia* and *Thileria* separately targeting a fragment of *18 S rRNA* gene of those parasites (Table 1). The thermal program for *Babesia* spp was as follows: initial incubation at 94 °C for 5 min, followed by 40 cycles of 94 °C for 45 s, 58 °C for 30 s, and 72 °C for 45 s, and a final extension at 72 °C for 7 min (Hilpertshauser et al. 2006; Khamesipour et al. 2015). The piroplasms positive samples were also examined for the presence of *Theileria* spp. using the relevant specific primer pairs (Table 1) targeting *18 S rRNA* gene. The PCR amplification conditions for *Theileria* spp were followed according to Barghash et al. 2016.

#### PCR screening of *Babesia bigemina* using specific primers

Depending on sequencing results, confirmed *Babesia* spp. positive samples using Bab-sp-F/R primers, were retested for the presence of *Babesia bigemina* using the specific primers targeting the *18 S rRNA* gene as shown in Table 1 and the amplification was carried out according to Barghash et al. 2016.

### Molecular detection of Anaplasmataceae

#### Conventional PCR targeting conserved region of 16 S rRNA gene to detect Anaplasmataceae

All ticks’ DNA samples were assayed by cPCR, using primers targeting the *16 S rRNA* gene, to detect *Anaplasma* spp (Table 1). The PCR amplification conditions were applied according to Cardoso et al. 2016; as follows: initial denaturation at 95 °C for 5 min; followed by 35 cycles of denaturation at 95 °C for 30 s; annealing and extension at 65 °C for 30 s; 10 cycles at 60 °C for 30 s; and final extension at 72 °C for 30 s. After the last cycle, the extension step was extended for a further 5 min. A tick sample known as positive for *Anaplasma* spp. was used as a positive control (Abdel-Shafy et al. 2022) and negative control (lack of DNA) was included also in each reaction.

#### PCR screening of*Anaplasma marginale* and *Wolbachia* spp. using specific primers

Depending on the sequencing results, samples that were verified positive for Anaplasmataceae were retested for the presence of *Anaplasma marginale* according to Kundave et al. (2018) and *Wolbachia* spp according to Laidoudi et al. (2020), using specific primers targeting *16 S rRNA* gene and *ftsZ* gene, respectively, in cPCR and the primer sequences are shown in Table 1.

**Table 1:** DNA sequences of the used primers

Species	Gene name	Primer Forward	Primer Reverse	Size of PCR product (bp)	Annealing Temperature °C	Reference
*H. dromedarii*	*CO1*	**F** GGAACAATATATTTAATTTTTGG	**R** ATCTATCCCTACTGTAAATATATG	850	55	Chitimia et al. 2010
Piroplasms	*18 S rRNA*	**RLB-F2** GACACAGGGAGGTAGTGACAAG	**RLB-R2** CTAAGAATTTCACCTCTGACAGT	390	External 52	Liu et al. 2016
		**FINT** GACAAGAAATAACAATACRGGGC		388	Internal 50	
*Babesia/Theileria* spp.	*18 S rRNA*	**F** AATACCCAATCCTGACACAGGG	**R** TTAAATACGAATGCCCCCAAC	400	58	Pereira et al. 2016
*Babesia* spp.	*18 S rRNA*	**Bab-sp‐F** GTTTCTGCCCCATCAGCTTGAC	**Bab-sp‐R** CAAGACAAAAGTCTGCTTGAAAC	422–440	58	Hilpertshauser et al. 2006; Khamesipour et al. 2015
*Theileria* spp.	*18 S rRNA*	**989 F** AGTTTCTGACCTATCAG	**990 R** TGCCTTAAACTTCCTTG	1100	54	Barghash et al. 2016
*B. bigemina*	*18 S rRNA*	TAGTTGTATTTCAGCCTCGCG	AACATCCAAGCAGCTAHTTAG	639	60	Barghash et al. 2016
Anaplasmataceae	*16 S rRNA*	**ECB**: CGTATTACCGCGGCTGCTGGCA	**ECC**: AGAACGAACGCTGGCGGCAAGC	500.	65	Rufino et al. 2013; Cardoso et al. 2016
*A. marginale*	*16 S rRNA*	**Amar16S-F**: GGCGGTGATCTGTAGCTGGTCTGA	**Amar16S-R** GCCCAATAATTCCGAACAACGCTT	270	55	Kundave et al. 2018
*Wolbachia sp.*	*ftsZ*	**Wol.ftsZ.363.F** GGRATGGGTGGTGGYACTGG	**Wol.ftsZ.958.R** GCATCAACCTCAAAYARAGTCAT	560	55	Laidoudi et al. 2020

### Sequence analyses

Representative number of strong positive amplifications of the target genes of *H. dromedarii* ticks, *Babesia*/*Theileria* spp., *Babesia* spp., Anaplasmataceae, *B. bigemina*, *A. marginale* and *Wolbachia* sp., were purified using the GeneDirex PCR clean-up and Gel Extraction kit (Taiwan) according to the manufacturer’s instructions and sent for bi-directional Sanger sequencing (Macrogen -Seoul, South Korea) using ABI3730XL DNA Sanger sequencer (ThermoFisher) (Waltham, MA, United States). The basic local alignment search tool (BLASTN) was used for species identification of the obtained sequences. The sequencing results were aligned with reference sequences and edited using MEGA7 software (https://www.megasoftware.net/download_form). Query cover and the identity percentage among the compared sequences were calculated by NCBI and clustal omega website (https://blast.ncbi.nlm.nih.gov/Blast.cgi) and (https://www.ebi.ac.uk/Tools/msa/clustalo/), respectively. After the comparison with the different isolates, the resulted sequences of the detected *Babesia/Theileria* spp., *Babesia* spp. and Anaplasmataceae Egyptian isolates in *H. dromedarii* ticks were submitted to GenBank.

### Phylogenetic analysis

To assess the genetic diversity of hemoparasites within the study samples, species specific phylogenetic trees were constructed using phylogenetic tree prediction generated by MEGA7 (https://www.megasoftware.net/download_form). The phylogenetic analysis was carried out using the Maximum Likelihood method based on the Kimura 2-parameter model (Kumar et al. 2016). The target gene sequences of *H. dromedarii* tick as well as *Babesia/Theileria* spp., *B. bigemina*, *A. marginale* and *Wolbachia* sp. detected in *H. dromedarii* ticks and different reference sequences in GenBank were used for comparative molecular analysis. The *Ixodes ricinus* (accession number: AJ300195.1) was included as an outgroup in the tree of *H. dromedarii* tick. Cytochrome oxidase of *Eimeria* sp. parasite (accession number: KT305929.1) (Al-Habsi et al. 2017) and *B. bovis* parasite ribosomal protein L12eI (accession number: M81359.1) (Dalrymple and Peters 1992) were included in the trees of pathogens as outgroups.

### Statistical analysis

The chi-square (χ^2^) test was applied at a probability of *p* < 0.05 to compare infection rates by piroplasms, *Babesia* spp. and Anaplasmataceae among different months. Significant associations were identified when a *p* value of less than 0.05 was observed (Snedecor and Cochran 1989).

## Results

### Molecular identification of *H. dromedarii* tick

All PCR screened *H. dromedarii* samples, using specific primers of *CO1 *DNA marker, resulted in an amplification of 850 bp fragment which is consistent with the expected documented length of *CO1* gene.

### Detection of piroplasms in *H. dromedarii* ticks

**Fig. 1:**
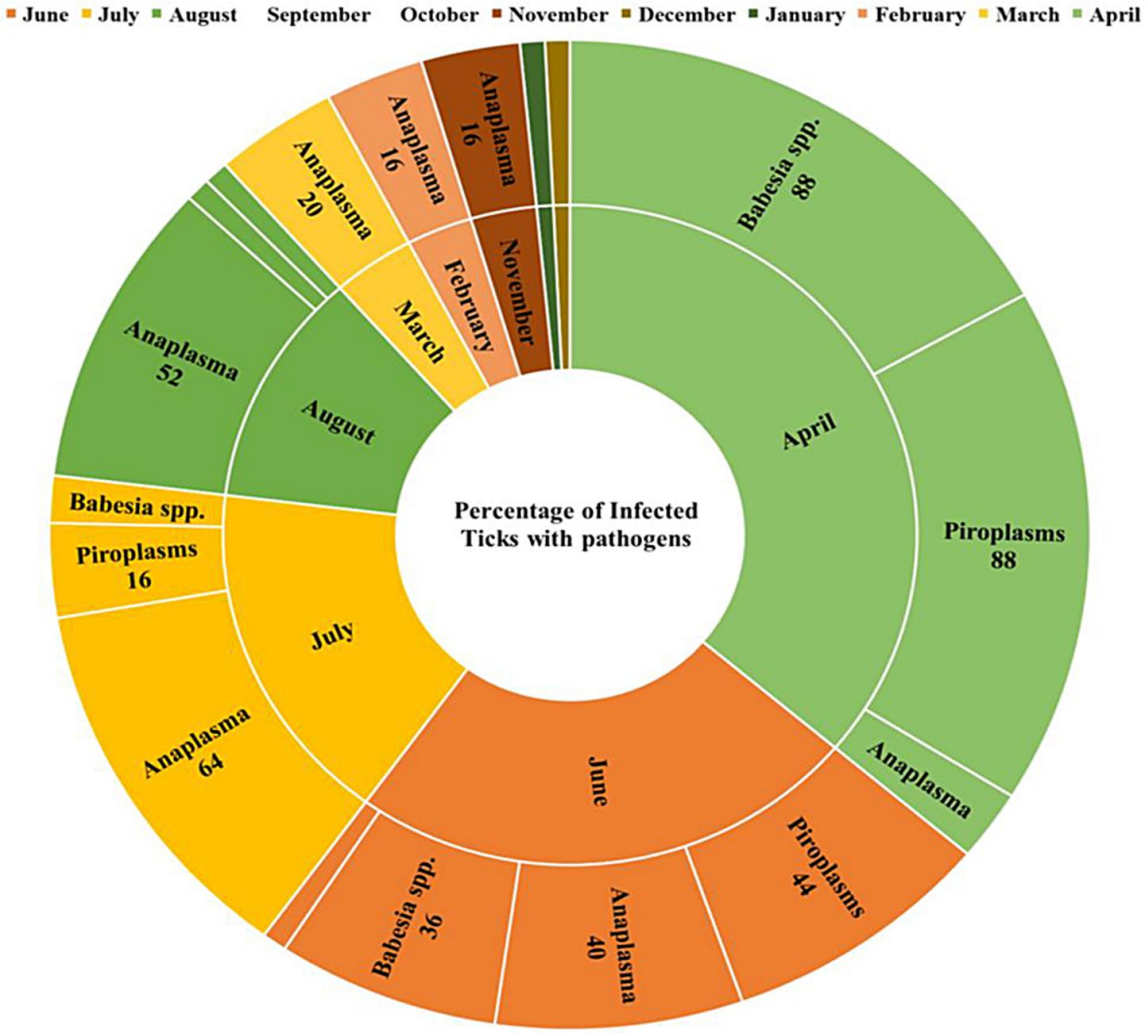
The percentage of piroplasms, *Babesia* spp., *Theileria* spp. and Anaplasmataceae detected monthly by PCR in *H. dromedarii* semi-engorged females (June 2021 to April 2022)

The semi-nPCR results targeting *18 S rRNA* gene revealed that the overall rate of piroplasms in semi-engorged females of *H. dromedarii*, from all seasons, was 13.8%. The monthly rate of piroplasms was 44%, 16%, 4% and 88% in June-, July-, August-2021 and April 2022, respectively. Piroplasms were not detected in the samples collected from the remaining months as shown in Table 2. Statistically, there was a significant difference in the rate of piroplasms infection among different months based on semi-nPCR data (*p* < 0.05) as shown in Table 2; Fig. 1. The positive samples showed the expected amplicon size at 388 bp in the internal PCR reaction.

### Detection of *Babesia* spp. DNA in *H. dromedarii* ticks

To detect *Babesia* spp., all piroplasms positive tick samples were screened by PCR targeting *18 S rRNA* gene. Out of 38 piroplasms positive ticks, 35 ticks (92.1%) were positive to *Babesia* spp., recording a general rate of 12.7% (35/275). The positive samples revealed the expected amplicon size (422–440 bp). *Babesia* spp. was detected in ticks in June, July, August, and April with rate of 36%, 8%, 4% and 88%, respectively (Table 2). Statistically, there was a significant difference in the rate of *Babesia* spp infection among different months based on cPCR data (*p* < 0.05) as shown in Table 2; Fig. 1.

### Detection of *Theileria* spp. DNA in *H. dromedarii* ticks

To detect *Theileria* spp., all positive tick samples for piroplasms were screened by PCR targeting *18 S rRNA* gene. Only one positive *Theileria* spp. tick (4%) was detected in June with a general rate of 0.4% (1/275) (Table 2; Fig. 1). The positive sample gave the expected amplicon size of 1100 bp.

### Detection of Anaplasmataceae in *H. dromedarii* ticks

The cPCR results targeting *16 S rRNA* gene revealed that the overall rate of Anaplasmataceae was 20.7%. The high rates of Anaplasmataceae were 40%, 64%, 52%, 16%, 16%, and 20% in June, July, August, November, February, and March, respectively (Fig. 1). Anaplasmataceae was not detected in the ticks collected in September and October (Table 2). Statistically, there was a significant difference in the rate of Anaplasmataceae in *H. dromedarii* ticks infection among different months based on cPCR data (*p <* 0.05) as shown in Table 2; Fig. 1. The positive samples showed the expected amplicon size of 500 bp.

**Table 2:** The number of *H. dromedarii* semi-engorged females infected with piroplasms, *Babesia* spp., *Theileria* spp. and Anaplasmataceae detected monthly by PCR (June 2021 to April 2022)

Month	Number of examined ticks	Number of Infected ticks			
		Piroplasms	Babesia spp.	Theileria spp.	Anaplasmataceae
June	25	11	9	1	10
July	25	4	3	0	16
August	25	1	1	0	13
September	25	0	0	0	0
October	25	0	0	0	0
November	25	0	0	0	4
December	25	0	0	0	1
January	25	0	0	0	1
February	25	0	0	0	4
March	25	0	0	0	5
April	25	22	22	0	3
Total	275	38	35	1	57
Chi square		27.474	30.714	-	20.000
*P* value		< 0.001	< 0.001	-	0.003

### Sequencing and phylogenetic analyses

**Fig. 2:**
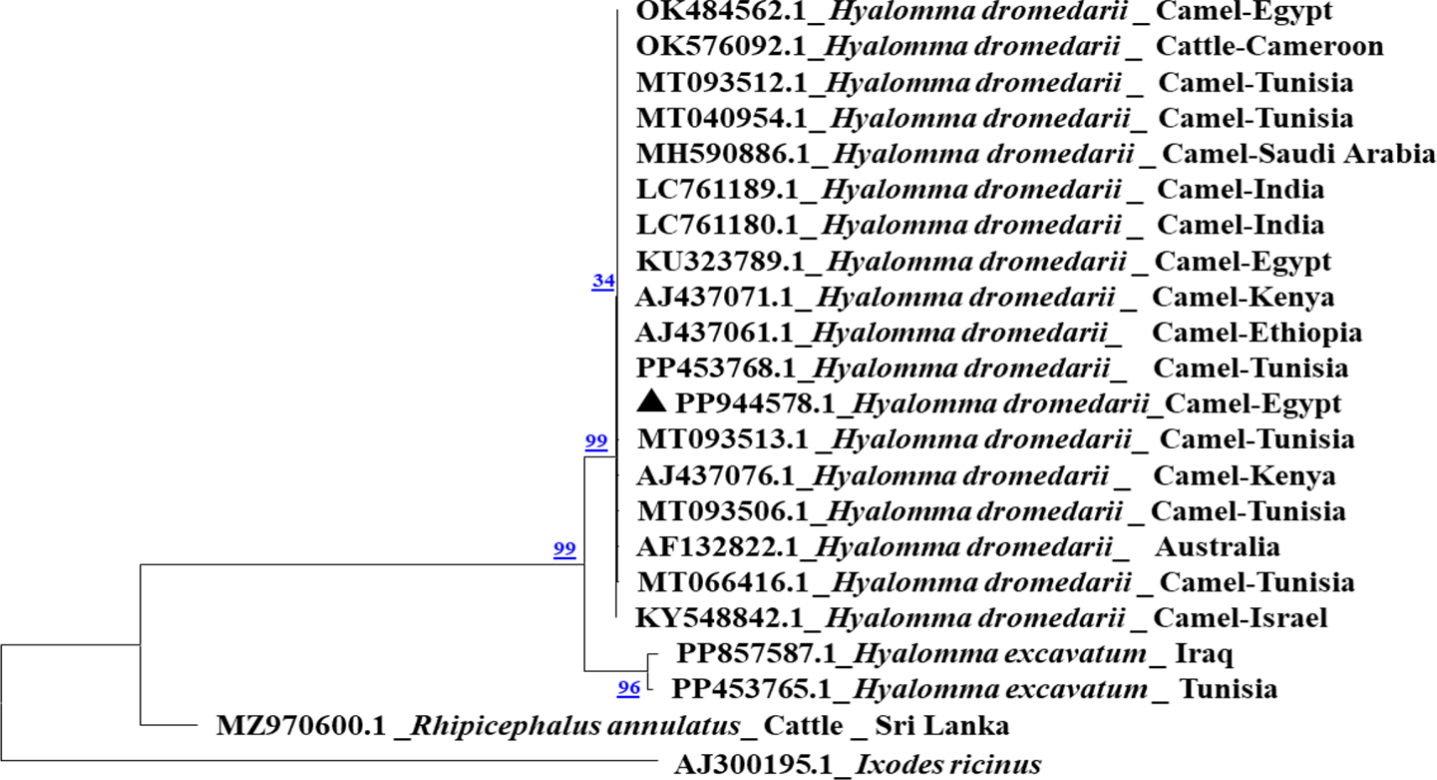
Phylogenetic analysis by Maximum Likelihood method of *H. dromedarii CO1* gene. The sequence is labelled with triangle and showed 100% similarity with other reference sequences. The *Ixodes ricinus* *16 S rRNA* gene was used as an outgroup

To confirm the molecular identification of *H. dromedarii* and parasite identity, the obtained sequences were subjected to Basic local Alignment Search Tool (BlASTN). All the resulting sequences of the amplified target gene fragments were deposited in GenBank under accession numbers: PP944578.1 (*H. dromedarii*), OQ932865.1-OQ932869 (*B. bigemina*), OQ932863.1 (*A. marginale*) and OQ932864.1 (*Wolbachia* sp.). BlastN analysis revealed that the *H. dromedarii CO1* gene sequenced samples have identity percent of 100% to *H. dromedarii* infesting camels from Tunisia, Kenya, India and Saudi Arabia (Fig. 2). Moreover, the nucleotide sequences of the target genes of *Babesia/Theileria* spp. positive samples (*n* = 7) have identity percent of 89% and query coverage of 100% to *B. bigemina* isolates from Mexico, Egypt and Turkey from cattle origin, and USA from cattle ticks. Moreover, blast analysis of *Babesia* spp. positive samples (*n* = 7) revealed that all sequenced samples have identity ranging from 98 to 100% and query coverage of 100% to *B. bigemina* isolates from Egypt and Turkey from cattle origin.

The sequence of Anaplasmataceae-identifying gene fragment detected in *H. dromedarii* ticks (6 selected positive samples from different months) showed relatively distinct isolates compared to those deposited in NCBI databases. Four positive samples of Anaplasmataceae showed similarity between 88 and 91% and 94–100% of query coverage to previously published sequences of *16 S rRNA* gene of *A. marginale* isolates from ticks originated from Nigeria and Egypt. The other 2 selected positive samples from different months showed identity of 86% with 100% query coverage to previous published sequences of *16 S rRNA* gene of *Wolbachia* spp. isolates from Italy.

### The prevalence of *B. bigemina*, *A. marginale* and*Wolbachia* sp

*Babesia bigemina* was detected in 7 samples out of 38 samples that tested positive for *Babesia* spp. while *A. marginale* and *Wolbachia* sp were detected in 27 and 4 samples, respectively out of 57 samples that tested positive for Anaplasmataceae.

### Sequencing and phylogenetic analyses of *B. bigemina*,* A. marginale* and *Wolbachia* sp.

**Fig. 3:**
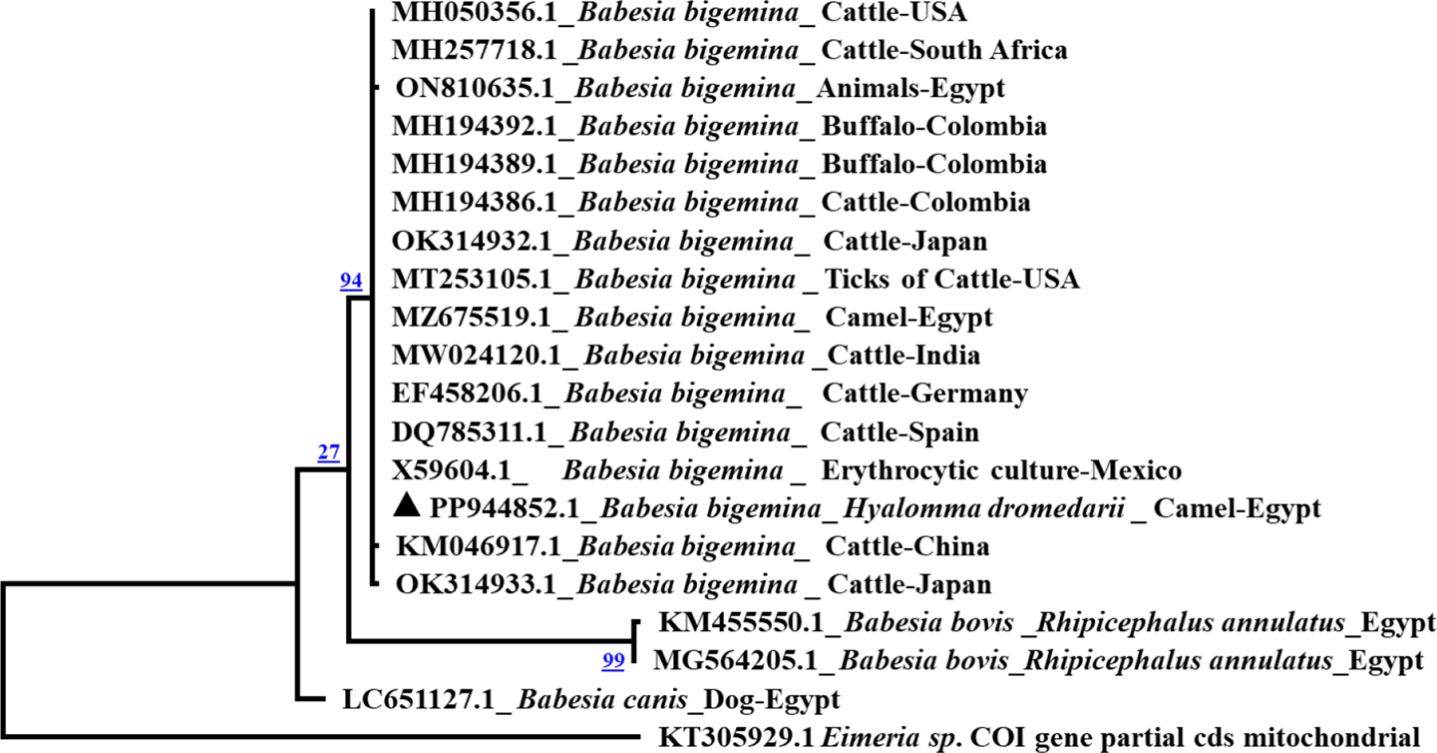
Phylogenetic analysis by Maximum Likelihood method of four *B. bigemina* *18 S rRNA *gene sequences detected in *H. dromedarii* ticks. The sequence is labelled with triangle and showed similarities between 99.2–99.8% with other reference sequences. Cytochrome oxidase gene from *Eimeria* sp. was used as an outgroup

**Fig. 4:**
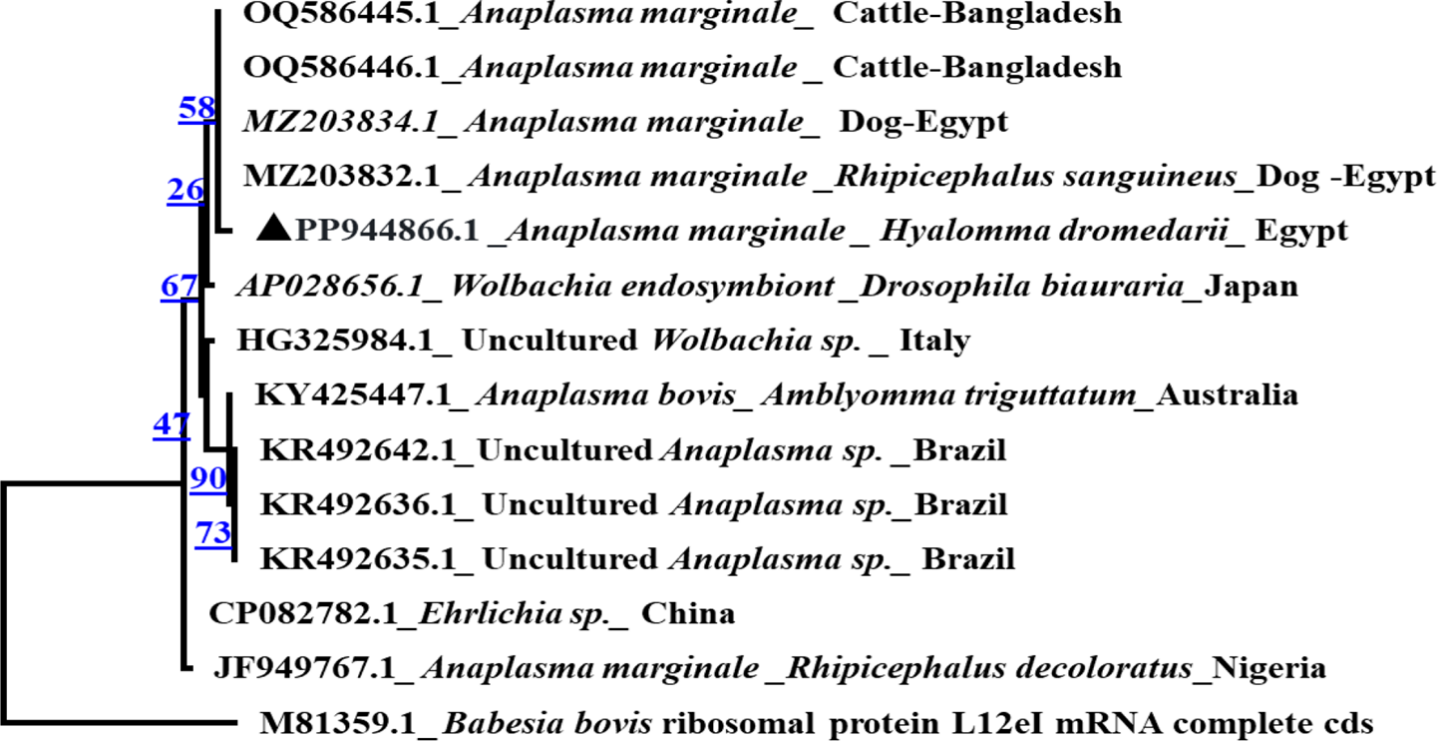
Phylogenetic analysis by Maximum Likelihood method of *A. marginale* *16 S rRNA * gene detected in *H. dromedarii* ticks. The sequence is labelled with triangle and showed 94% similarity with other reference sequences. The ribosomal protein L12eI gene from *B. bovis* was used as an outgroup

**Fig. 5:**
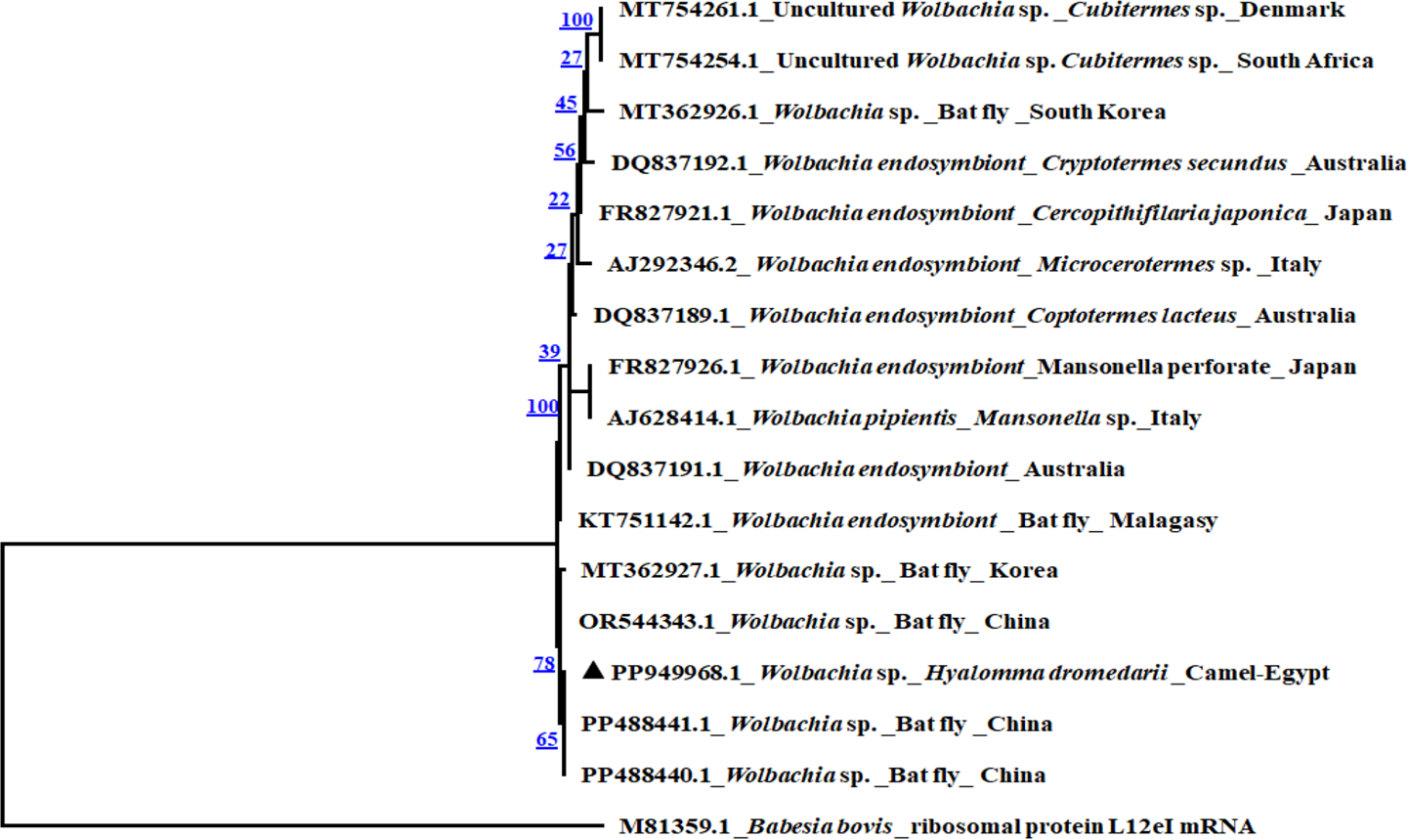
Phylogenetic analysis by Maximum Likelihood method of *Wolbachia* sp *ftsZ* gene detected in *H. dromedarii* ticks. The sequence is labelled with triangle and showed 99.7% similarity with other reference sequences. The ribosomal protein L12eI gene from *B. bovis* was used as an outgroup

The BlastN analysis of *B. bigemina* (*n* = 2) under accession number PP944852.1, showed that the present study sequences have identity percent of 99.8% to *B. bigemina* from cattle in USA and South Africa and *B. bigemina* from camel in Egypt in GenBank databases. Moreover *A. marginale* sequences (*n* = 2) under accession number PP944866.1 revealed high similarity (94%) to *(A) marginale* from cattle in Bangladesh. In addition, *Wolbachia* sp sequence (*n* = 2, accession number PP949968.1) showed similarity of 99.7% with *Wolbachia* sp. in bat fly from China and Korea. The phylogenetic analysis showed that *(B) bigemina* Egyptian isolate in the present study was clustered with *B. bigemina* isolates from South Africa, Colombia, Egypt, Japan and India in the same clade (Fig. 3). Moreover, *A*. *marginale* Egyptian isolate in the present study was clustered with *A. marginale* isolates in *R. sanguineus* and dog from Egypt and *A. marginale* isolates from cattle in Bangladesh in the same clade (Fig. 4). In addition, *Wolbachia* sp Egyptian isolate was clustered with *Wolbachia* sp. in bat fly from China in the same clade (Fig. 5).

## Discussion

Climate changes affect worldwide biodiversity and distribution of arthropods and arthropod-borne diseases. The weather in Egypt became warm throughout the year and the ticks can be found during all seasons of the entire year with high tick population in the summer and lower tick number in the winter (El-Sayed and Kamel 2020). In Egypt, camels are mainly infested by *H. dromedarii* beside other tick species belong to the genus *Hyalomma* such as *H. anatolicum*, *H. impeltatum*, *H. rufipes*, *H. turanicum* (Abdel-Shafy 2008a,b; Abdel-Shafy et al. 2016; Barghash et al. 2016). Recent studies focused on screening the presence of haempathogens in camels but omitted the tick vectors except for some few studies that investigated a random small number of ticks. In general, there is limited information available about the rate of piroplasms and Anaplasmataceae infection in the camel tick *H. dromedarii* in Egypt, especially among different seasons of the year. So, to evaluate the status of piroplasmosis and anaplasmosis in Egypt, we investigated the infections of *Babesia*, *Theileria* and Anaplasmataceae in *H. dromedarii* ticks collected from camels in Egypt over seasons of a year starting from June 2021 to April 2022.

This study hypothesizes that the detection of blood pathogens in camel ticks indicates that camels or other hosts might be infested by *H. dromedarii* which serves as a natural reservoir of pathogens. More probably, these pathogens could be transmitted to other susceptible surrounding hosts like cattle, equine and sheep residing in endemic areas especially during high tick infestation season. Molecular piroplasms investigation in our work indicated that there were 38 positive samples for piroplasms, 35 of them were *Babesia* spp and 1 sample was identified as *Theileria* spp. The remaining two piroplasms positive samples might be one of the other piroplasms as *Cytauxzoon* (Schnittger et al. 2022). In a recent study conducted in Egypt by Hassan et al. (2024) based on PCR, it was observed that, 386 out of 961 cattle were positive for piroplasms DNA with a prevalence rate of 40.16%, and authors record 114 (11.9%) cattle were infected with *Theileria annulata*.

The idea of detection of piroplasms in different tick species other than *H. dromedarii* was a point of research and was previously performed through few recent studies (Ngnindji-Youdje et al. 2022; Jaenson et al. 2024; Obaid et al. 2024; Springer et al. 2024). In our work, the molecular results indicated that piroplasms were detected in 38 (13.8%) of *H. dromedarii* samples. This result is higher than that reported by Ngnindji-Youdje et al. 2022) in Cameroon, who detected that the rate of piroplasms was 7.3% in different tick genera and species from cattle (*R. microplus*; *R. lunulatus*; *Amblyomma variegatum*, *H. rufipes* and *Haemaphysalis leachi*). The highest rate of piroplasms, in the present study, was in spring (April) followed by summer (June and July) however, piroplasms were not detected in the ticks collected from the remaining months. Probably that returns to the high tick activity during these months since high tick infestation in Egypt occurs during spring and summer seasons as reported by Diab et al. (2001) and Barghash et al. (2016). The high tick activity in these seasons is due to favorable environmental conditions such as high temperature and humidity rather than the rainy ones. In this context, our results are consistent with Mahdy et al. 2023), who mentioned that the highest rate of camel piroplasmosis in Egypt was in spring followed by summer. Nevertheless, we found that the rate of piroplasms was very low in August although it belongs to the summer season. Consequently, we may refer piroplasms low abundance in August, sometimes to aridity which may force ticks to undergo latency to avoid loss of energy, which may be exacerbated too by pathogen infections (Reye et al. 2010). The samples that were positively identified by nPCR, for piroplasms, were examined by cPCR using primers specific for either *Babesia* spp. or *Theileria* spp. to detect the samples that test positive for these two species only. The rate of *Babesia* spp. in this study was 12.7% which was higher than *Theileria* spp. (0.4%). This finding is not in agreement with Barghash et al. (2016) in Egypt, who recorded *Babesia* spp. rate of 45.5% compared to 75.8% of *Theileria* spp. in *Hyalomma* spp. (*H. dromedarii*,* H. rufipes*,* H. truncatum*,* H. excavatum*,* H. impeltatum*) collected from camels. Herein, the higher rate of *Babesia* spp. over *Theileria* spp. may be due to collecting ticks from camels raised with cattle in the same area or farm. On the contrary, we detected higher rate of *Babesia* spp. than that mentioned by Thankgod et al. (2020) in Nigeria (0.7%), where *H. dromedarii* and *H. impeltatum* are infesting camels. This overall variation may be due to the difference in tick species that were examined, season of sampling and the geographical areas comprising camel habitats.

Representative number of *Babesia*/*Theileria* spp. positive samples (*n* = 7) by cPCR in addition to *Babesia* spp. positive samples with strong bands (*n* = 7) were sequenced to confirm the presence of *Babesia* spp. DNA in *H. dromedarii*. Blast analysis of NCBI revealed that the obtained sequences were related to *B. bigemina.* This result agrees with Barghash et al. (2016) who detected *B. bigemina* in the examined camel tick samples in Egypt (*H. dromedarii*,* H. rufipes H. truncatum*,* H. excavatum*,* H. impeltatum*). Also the results are consistent with Palomar et al. (2022) who detected *B. bigemina* in *Rhipicephalus (Boophilus) decoloratus* ticks which are distributed in Benguela city (Angola).

Depending on the sequencing results, we used specific primers for *B. bigemina* in cPCR to detect its rate in *H. dromedarii* samples. The rate of *B. bigemina* in the present study was 2.5% which is lower than the reported data by Barghash et al. (2016) in Egypt, who detected *B. bigemina* in camel ticks with rate of 27.2%. Adham et al. (2009) identified a 66% rate for *B. bigemina* in *Rhipicephalus (Boophilus) annulatus* (*R. annulatus*) ticks. However, other studies showed lower rate of *B. bigemina* such as the work carried out by Hassan et al. (2017) in Egypt who recorded 0.9% of the pathogen in *R. annulatus* and 2% in *Rhipicephalus bursa* collected from various domestic and wild hosts in Corsica (France) (Grech-Angelini et al. 2020). These variations are possibly related to several factors including tick species, number of samples, season, geographical location, diagnostic approaches and feeding status of the ticks. Representative number of the samples tested positive for *B. bigemina* were sequenced and the blast analysis of the present study sequences, showed that *B. bigemina* Egyptian isolate from *H. dromedarii* under accession number PP944852.1 has identity percent of 99.8% to *B. bigemina* from cattle in USA (MH050356.1) and South Africa (MH257718.1) and *B. bigemina* from camel in Egypt (MZ675519.1).

The molecular identification results were consistent with previous epidemiological data related to blood parasites in camels, which is broadly related to the presence and distribution of their vectors (Barghash et al. 2016). Camels could be infected with different blood parasites such as *Babesia* spp. (*B. bovis*, *B. bigemina* and *B. microti*) (Naga and Barghash 2016; Rizk 2021; El-Alfy et al. 2022; Salman et al. 2022; Mahdy et al. 2023; Ashour et al. 2023), and *Theileria* spp. (*T. annulata* and *T. equi*) (Naga and Barghash 2016; Mahdy et al. 2023), not only in Egypt but also in Sudan (Ibrahim and Kadle 2017), and Iran and Saudi Arabia (Swelum et al. 2014; Khamesipour et al. 2015).

*Hyalomma dromedarii* collected samples were examined also for the presence of Anaplasmataceae. The rate of Anaplasmataceae in *H. dromedarii* in our study was 20.7% which is lower than that detected in *H. dromedarii* by Choubdar et al. (2021) who showed rates of 68.1% in Iran and 35.3% in different tick genera and species from cattle (*R. microplus*; *R. lunulatus*; *Amblyomma variegatum*, *H. rufipes* and *Ha. leachi*) in Cameroon (Ngnindji-Youdje et al. 2022). The highest rate of Anaplasmataceae, in our study, was observed in the summer season followed by spring but the lowest rate was detected in winter. This high rate may be attributed to the high tick activity in these seasons leading to high tick infestation during spring and summer seasons as reported by Diab et al. (2001) and Barghash et al. (2016) in Egypt. These results are in agreement with Aziz et al. (2022) in Pakistan, who reported that the highest seasonal rate of anaplasmosis in goat was in summer and the lowest was in winter. Our results is nearly consistent with Selim et al. (2021) in Egypt, who showed the highest seasonal rate of anaplasmosis in cattle was at early summer and late spring and the lowest was in autumn. In September, Anaplasmataceae was not detected which may highlight the aridity forces driving ticks to undergo quiescence to avoid critical loss of energy, which may be worsened by pathogen infections (Reye et al. 2010). In addition to the fact that there is little or no transovarial transmission of the bacteria in *Hyalomma* spp. ticks (Moore et al. 2018), so bacterial transmission depends on capturing the infection from the infested host.

Random representative samples from each month that tested positive for anaplasmataceae were sequenced (*n* = 6). It is worth mentioning that *A. marginale* is an intracellular obligate pathogen that is transmitted mainly by ticks of the genus *Rhipicephalus*. We have detected this bacterium besides *Wolbachia* in *H. dromedarii* ticks and the infection rate was 22.6% which is opposite to the work carried by Ngnindji-Youdje et al. (2022) who could not detect the *A. marginale* in two species of the genus *Hyalomma* (*H. rufipes* and *H. truncatum*). In Cameroon, these authors had detected the bacterium *A. marginale* in genus *Rhipicephalus* (*R. microplus*, *R. sanguineus*,* R. annulatus*) and the infection rate was 19%. The other detected anaplasmataceae pathogen; *Wolbachia* spp., was found in *Haemaphysalis leachi* in Cameroon with a rate of 16.7% (Ngnindji-Youdje et al. 2022). In addition, the arthropod endosymbiont bacterium; *Wolbachia* spp. has been previously reported in *Ixodes ricinus* ticks, in Slovakia (Subramanian et al. 2012) and France (Moutailler et al. 2016). Furthermore, *Wolbachia* may influence their hosts’ reproductive biology through variety of interactions (Frédéric 2019). *Wolbachia* was identified in another arthropods, as mosquitoes (Maria Inácio da Silva et al. 2021) and reported in several mosquito species in Cameroon (Walker et al. 2021; Bamou et al. 2021). Also was identified in one *I. ricinus* in Algeria (Boucheikhchoukh et al. 2018). However, the transmission mechanism of *Wolbachia* in ticks and its impact on tick biology remains obscure (Ngnindji-Youdje et al. 2022).

*Anaplasma marginale* was identified in *H. dromedarii* ticks, in the present study, with infection rate of 9.8%, which is lower than that detected by Barghash et al. (2016) in Egypt. The current results are still lower than the rate of *A. marginale* in *H*. *excavatum* (28.5%) and *R. annulatus* (18%) in Egypt as reported by Al-Hosary et al. (2021). In addition, Grech-Angelini et al. (2020) Corsica (France), detected *A. marginale* (4%) in ticks collected from various domestic and wild hosts (*R. annulatus*, *R. bursa*, *H. marginatum*,* I*. *ricinus*,* Ha. punctata* and *R. sanguineus* sensu lato (s.l.). These observed variations could be attributable to the geographical diversity of infected ticks or the sensitivity of various primers. Representative *A. marginale* positive samples were sequenced and the phylogenetic comparative analysis of the present study sequence showed that *A. marginale* Egyptian isolate from *H. dromedarii* under accession number PP944866.1 was clustered in the same clade with *A*. *marginale* from cattle in Bangladesh (OQ586445.1 and OQ586446.1) and *A. marginale* from Dog and *R. sanguineus* in Egypt (MZ203834.1 and MZ203832.1). Moreover, *Wolbachia* sp. was detected in *H. dromedarii* ticks, in the present study, with prevalence of 1.5%. This prevalence is lower than that detected by Hu et al. (2020) in China, who detected *Wolbachia* sp. in mosquitoes with prevalence rate of 93.5%. Representative number of *Wolbachia* sp. samples were sequenced and the comparative phylogenetic analysis revealed that *Wolbachia* sp. sequence in the present study under accession number PP949968.1 was clustered in the same clade with *Wolbachia* sp. from bat fly in China (PP488440.1).

Finally, we detected mixed infection between piroplasms and Anaplasmataceae in 13/275 samples (5%). This is due to the fact that ticks can harbor many disease-causing agents and camels can have multiple concurrent infections (Barghash et al. 2016).

## Conclusion

In the present study, identification and genetic characterization of piroplasms and Anaplasmataceae in *H. dromedarii* collected from camels in Egypt were investigated. This study represents the first molecular detection of piroplasms, *B. bigemina*, *A. marginale* and *Wolbachia* sp. together in semi-engorged females of *H. dromedarii* ticks from four seasons over a year. Unfortunately, we could not carry out the collection of *H. dromedarii* ticks from different geographical areas in Egypt which may be a limiting factor in this work. However, the tick samples were collected from camels that were admitted to the slaughter house which receives animals from different governorates to be slaughtered. Moreover, the results shown herein are essential as a starting point to evaluate potential hazards by detecting piroplasms and other pathogens in different animals especially those feeding in close spatial proximity and their vectors infesting one or more hosts. We propose that this work will pave the way for further genotyping of pathogens and further prevalence and correlation studies in other areas in Egypt.

## Data Availability

We declare all data is being provided within this manuscript.
